# Neuroendocrine and Behavioral Consequences of Hyperglycemia in Cancer

**DOI:** 10.1210/endocr/bqaa047

**Published:** 2020-03-20

**Authors:** Juan H Vasquez, Jeremy C Borniger

**Affiliations:** 1 Department of Biology, University of Texas – San Antonio, San Antonio, Texas; 2 Cold Spring Harbor Laboratory, Cold Spring Harbor, New York

**Keywords:** hyperglycemia, glucose sensing, IL-6, STAT3, metabolism

## Abstract

A hallmark of cancer is the disruption of cellular metabolism during the course of malignant growth. Major focus is now on how these cell-autonomous processes propagate to the tumor microenvironment and, more generally, to the entire host system. This chain of events can have major consequences for a patient’s health and wellbeing. For example, metabolic “waste” produced by cancer cells activates systemic inflammatory responses, which can interfere with hepatic insulin receptor signaling and glucose homeostasis. Research is just now beginning to understand how these processes occur, and how they contribute to systemic symptoms prevalent across cancers, including hyperglycemia, fatigue, pain, and sleep disruption. Indeed, it is only recently that we have begun to appreciate that the brain does not play a passive role in responding to cancer-induced changes in physiology. In this review, we provide a brief discussion of how oncogene-directed metabolic reprogramming disrupts host metabolism, with a specific emphasis on cancer-induced hyperglycemia. We further discuss how the brain senses circulating glucose concentrations and how this process goes awry as a response to distant neoplastic growth. Finally, as glucose-sensing neurons control diverse aspects of physiology and behavior, we link cancer-induced changes in energy balance to neuroendocrine and behavioral consequences for the host organism.

It has long been known that cancer patients disproportionately display impaired glucose tolerance (1). In 1885, for example, Freund made the original observation that 62/70 (~89%) of cancer patients were spontaneously hyperglycemic (2). More recently, a study of 850 cases revealed that a hyperglycemic response (blood glucose > 200 mg/dL) was observed 3 times more frequently in patients with cancer than in age-matched controls (3), and about 70% of patients with pancreatic cancer exhibit impaired glucose tolerance with unknown etiology (4). Causal factors driving these phenomena have been difficult to differentiate given the heterogeneous nature of cancer, cancer treatments, dietary choices, and lifestyle factors among patients. Hyperglycemia in cancer has a “chicken or the egg” problem, where it is unclear what comes first, the malignancy or disrupted glucose metabolism. Indeed, patients with diabediabetes and poor glycemic control have a higher incidence of certain cancer types than the general population, although this link is not uniformly observed and changes as a function of disease duration (5-8). Reciprocally, circulating glucose concentrations in patients without diabetes also predict cancer mortality (9). How cancer itself reorganizes the metabolism of the host to facilitate its own growth and the health consequences of these processes are poorly understood. Cancer elicits progressive hyperglycemia throughout the course of malignant growth in several preclinical models, providing support for the notion that tumors reorganize host systemic metabolism independent of extraneous factors like stress, age, or diet (10,11).

Chronic elevations in circulating glucose can be detrimental to health. Part of this is due to the actions of advanced glycation end products (AGEs) (12), which form as a result of interactions between reducing sugars (eg, glucose) and amino groups in proteins, lipids, and nucleic acids (ie, the Maillard reaction). This consequence of hyperglycemia can influence a vast array of long-lived proteins, including collagen, myelin, tubulin, plasminogen activator, and fibrinogen, as well as complement C3 and C1q (13-16). Accumulation of AGEs can, through these various pathways, promote the progression of a variety of diseases, including cancer (17-19). Beyond AGEs, glucose itself can act as a signaling molecule with pleiotropic effects on neural circuits and behavior, as we discuss later. In this review, we will focus on how oncogene-initiated changes in cellular metabolism propagate to the tumor microenvironment, and then to the entire organism (Fig. 1). We further discuss how this cascade of events results in a spurious feedback loop where glucose-sensing neurons in the brain aberrantly promote further hepatic gluconeogenesis to drive neuroendocrine and behavioral abnormalities in cancer.

**Figure 1.**
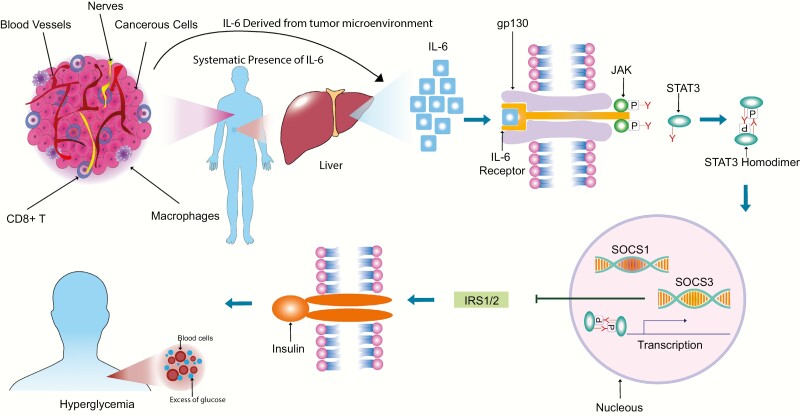
Propagation of inflammatory signaling from the tumor microenvironment drives systemic hyperglycemia in cancer. Changes in cellular metabolism result in the accumulation of metabolic “waste” within the tumor microenvironment (including lactate). These metabolites polarize local immune cells towards a phenotype that results in IL-6 release. Sufficient stimulation causes systemic concentrations of IL-6 to rise. Classical IL-6 receptor signaling within the liver (coupled to gp130) results in downstream transcription of STAT3 target genes, including SOCS1 and SOCS3. These gene products directly interact with insulin receptor substrates 1 and 2 (IRS1/2) and targets them for degradation. This results in impaired insulin-dependent glucose uptake and systemic hyperglycemia.

## Cancer metabolism: from cells to systems

Cancer develops as a result of the accumulation of oncogenic mutations that disrupt normal checkpoints in cell division and replication. Common mutational signatures across diverse types of cancer promote aberrant fuel utilization by the neoplastic cells themselves. This initial discovery, known as the Warburg effect, underlies the propensity of cancer cells to uptake glucose and ultimately produce elevated amounts of lactic acid through glycolysis, as opposed to oxidative phosphorylation (20,21).

Fuels such as sugars, fats, and amino acids are taken up by the cell in order to produce energy in the form of adenosine triphosphate (ATP) along with macromolecules such as deoxyribonucleic acid, ribonucleic acid (RNA), and proteins. Normally, these components are used to facilitate the development and proliferation of cells in a controlled fashion. Because cancer cells and healthy cells compete for the use of fuels, it follows that identification of the preferred metabolic pathways that tumors use could be useful in identifying cancer-specific metabolic vulnerabilities.

Aberrantly activated oncogenes and dysregulation of tumor suppressors alters the import and utilization of glucose and amino acids into cancer cells. This ultimately leads to the production of “waste” products such as lactate through glycolysis and changes in the use of the citric acid cycle intermediates for biosynthesis. For example, *c-myc*, a master regulator of transcription frequently overexpressed in cancer, increases the expression of lactate dehydrogenase A (LDHA), an enzyme which catalyzes the conversion of pyruvate into lactate (22). The abnormal consumption of glucose by tumors is used as a valuable tool for cancer monitoring via the use of positron emission imaging tomography. Radiolabeled ^18^F-fluorodeoxyglucose is used as a glucose tracer to target areas of abnormal glucose uptake (23,24). This technique has effectively allowed the localization of tumors with a high success rate (25). Lactate is known to be a byproduct that emerges from cancer cells after glycolysis, which is also consumed by mutated cells and serves as a fuel for the proliferation of tumors (21,26-28).

Due to observations that hypoxia-triggered stabilization of hypoxia inducible factor (HIF)-1α also initiates the upregulation of LDHA, it was thought that lactate primarily served hypoxic regions of the tumor (29,30). However, recent research has demonstrated that this statement is not entirely accurate (31). Lactate also serves as a substrate to fuel oxidative metabolism in oxygenated compartments of the tumor. This was observed after identification of the monocarboxylate transporter 1 as the preferred path for the transport of lactate in human cervix squamous carcinoma cell lines. Confirmation of this was provided through inhibition experiments, where blockade of monocarboxylate transporter 1 trough α-cyano-4-hydroxycinnamate (or small interfering RNA) resulted in a switch to glycolysis instead of lactate-fueled respiration (32). The ability of tumor cells to adapt to process glucose through glycolysis in both aerobic and anaerobic environments is of relevance since this allows the production of lactic acid, which further supports tumor maintenance and proliferation (33).

Lactate is an important component that links the tumor microenvironment to systemic metabolic processes. The presence of lactate promotes complex immune responses within local immune cells. For example, excess lactate in the extracellular space promotes the emergence of a permissive environment for continued tumor growth due to the attenuation of dendritic and T-cell activation, as well as monocyte migration (34-36). Additionally, lactate can polarize macrophages in the tumor microenvironment to an alternatively activated M2 state associated with wound healing and tissue repair (37). Conversely, lactate stimulates interleukin (IL) 23 production, which is proinflammatory and promotes inflammation in tumor microenvironments (38). Induced overproduction of IL-23 is known to be linked to angiogenesis and the progression of cancer, metastasis, and resistance to chemotherapy in diverse carcinomas (39). In tumor environments, the expression of the cytokine interleukin 23 is widely observed (40). Other pleiotropic cytokines that share downstream signaling pathways with IL-23, like IL-6, are also induced through immunometabolic interactions in the tumor microenvironment (41). Specifically, tumor-derived lactate polarizes local myeloid cells toward an M2 phenotype, boosting IL-6 release (42). Sustained elevations in IL-6 propagate into systemic circulation and activate “classical” IL-6 receptors (IL-6Rα) in the liver. This is a critical link between ongoing inflammatory processes in the tumor microenvironment and systemic metabolic effects of cancer.

In the liver, IL-6 acts as a general alarm and induces a coordinated network of physiological changes termed the “acute phase response,” in order to restore homeostasis. During chronic inflammatory conditions, like cancer, this process becomes hijacked and hepatic function can breakdown (43,44). This is a result of the actions of downstream components in the IL-6 signaling cascade. In classical IL-6 signaling, binding of its gp130-coupled membrane-bound receptor (IL-6Rα) activates janus kinase (JAK)/signal transducer and activator of transcription 3 (STAT3) signaling. Phosphorylated STAT3 translocates to the nucleus to drive the expression of hundreds of target genes (45). One of these genes, Suppressor of cytokine signaling-3 (*SOCS3*), acts as a negative regulator on sustained STAT3 signaling through its interactions with JAK kinase and cytokine receptors (46). This normally acts to terminate the acute phase response. During sustained inflammation, however, the overexpression of SOCS proteins occurs. SOCS1 and SOCS3 directly affect insulin receptor substrates (IRS-1/2) by targeting them for ubiquitination-mediated degradation (47). This has extensive consequences for systemic glucose metabolism. Because IRS-1/2 are critical components in the insulin signaling pathway, which is responsible for the insertion of glucose transporters into cell membranes (specifically GLUT4), their destruction results in impairments in glucose uptake from circulation. Therefore, unchecked IL-6/STAT3/SOCS3 signaling driven by cancer-derived metabolic waste directly impairs insulin signaling to promote hyperglycemia.

## Glucose-sensing neurons and the consequences of unchecked hyperglycemia

Changes in host metabolism are under homeostatic control by the brain to ensure energy balance is maintained in the face of environmental and physiological challenges. As glucose is the predominant source of fuel for neurons, the brain must keep a careful watch on local and systemic glucose concentrations. Oncogene-directed changes in nutrient uptake, reprogramming of intracellular metabolism, and subsequent secretion of inflammatory metabolic “waste” all contribute to changes in systemic energy balance via complex and poorly understood mechanisms, some of which we discussed above (48). Several subcortical nuclei play a major role in detecting and responding to changes in metabolic factors in the periphery. We discuss several of these below, but refer the reader to additional resources for more detail on neural control of energy balance, as discussing all relevant populations is beyond the scope of this review (49-53).

**Figure 2.**
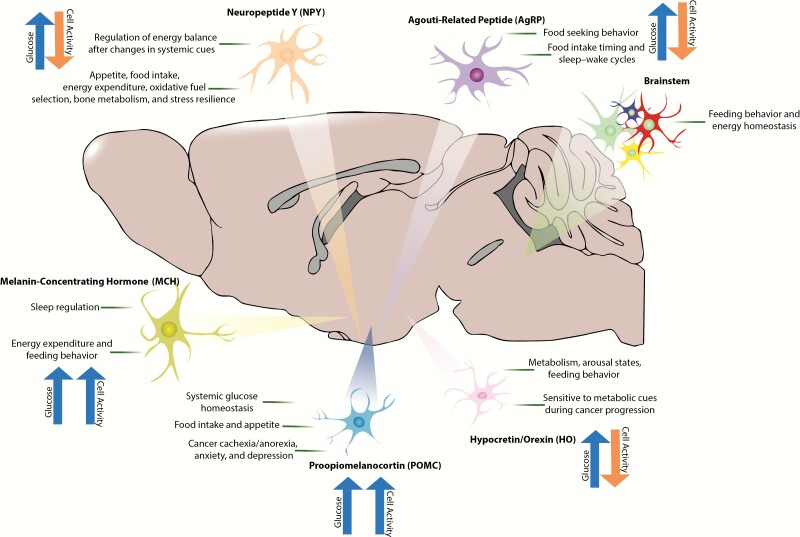
Glucose-sensitive neuronal populations putatively altered in the context of cancer-induced hyperglycemia. Several populations of neurons within the hypothalamus and brainstem are sensitive to extracellular changes in glucose concentrations. These neurons control a vast array of behavioral and physiological functions, including energy balance, sleep/wake states, feeding behavior, stress resilience, and hepatic gluconeogenesis, among other functions. Therefore, cancer-induced changes in glucose likely has far-reaching consequences on central neuronal activity and subsequent physiology/behavior. Understanding and manipulating these circuits may provide a novel approach for treating cancer-associated co-morbidities including sleep disruption, fatigue, cachexia/anorexia, depression, and anxiety.

Specialized subsets of brainstem and hypothalamic neurons show specific responses (inhibitory or excitatory) to changes in extracellular glucose concentrations (for review, see (54,55)). Careful coordination between these 2 brain regions is essential for appropriate regulation of systemic physiology (56-58). For a long while it was assumed that most glucose-sensitive neurons used a sensing strategy similar to that of pancreatic β cells, where glucose-induced elevations in intracellular ATP results in the closure of potassium (K^+^) permeable K_ATP_ channels, resulting in depolarization and excitation. However, work in the early 21st century demonstrated that, while this mechanism holds true for a few neural populations, many employ different and unique strategies to sense and respond to glucose. Discovered in the 1960s, hypothalamic glucose-sensing neurons were found to reside in the lateral, arcuate, and ventromedial hypothalamic nuclei (59), while those in the brainstem were distributed across the area postrema, nucleus of the solitary tract, and dorsal motor nucleus of the vagus. Importantly, these neurons play vital roles in physiological homeostasis, sleep–wake control, feeding behavior, and other processes that become disrupted in cancer. Therefore, tumor-induced hyperglycemia has far-reaching consequences beyond energy balance that impair quality of life for patients with cancer (Fig. 2). Additionally, many hypothalamic neuronal populations send reciprocal outputs to regulate hepatic gluconeogenesis. Indeed, this was initially observed in the 1970s in experiments where liver function was determined in rats receiving electrical stimulation of the ventromedial and lateral hypothalamus (60). Since these initial experiments, progress has been made in understanding the mechanisms of hypothalamic control of hepatic gluconeogenesis (61-64). Although a detailed discussion of this phenomenon is beyond the scope of this review, it is relevant when thinking about feedback loops between cancer-induced changes in systemic metabolism and top-down regulation of hepatic function driving hyperglycemia.

## Hypocretin/orexin

Neurons in the lateral hypothalamus expressing the excitatory neuropeptides hypocretin-1 and -2 (orexin-A and -B) serve a fundamental role in coupling metabolic and arousal states (49,65-69). They sense a wide variety of systemic metabolic cues that become deregulated during cancer progression, including glucose, leptin, ghrelin, pH and carbon dioxide, cholecystokinin, amino acids, and glucocorticoids, among others (70). In regard to our current discussion, hypocretin/orexin (HO) neurons serve as both sensors and regulators of glucose metabolism via multiple mechanisms. First, they are directly inhibited by physiological elevations in extracellular glucose concentrations through actions of a tandem-pore potassium channel (K_2P_), which ultimately changes membrane potential and neuronal excitability in response to changes in metabolite concentrations (55,71,72). This mechanism is unique among other glucose-sensing neurons, which usually use a “β cell strategy,” relying on glucokinase and/or ATP-sensitive potassium channels (73). K_2P_ channels, which carry a leak potassium current, are additionally sensitive to changes in pH and oxygen (74), providing HO neurons with diverse sensing capabilities.

In a reciprocal fashion to that described above, HO neurons depolarize and fire in response to insulin-induced hypoglycemia (75-77). An ultimate explanation for this bidirectional control of HO neurons by physiological concentrations in glucose is to promote rest and recovery in response to positive energy balance (high extracellular glucose) and arousal and food-seeking in response to periods of negative energy balance (low extracellular glucose). This mechanism links changes in metabolic state with arousal, as HO neurons regulate the activity of diverse neuronal systems involved in both sleep/wake states and energy balance. For example, HO neurons exert their arousal-promoting activities via direct synaptic connections onto locus coeruleus noradrenergic neurons (78,79), and they influence feeding behavior via similar inputs to proopiomelanocortin (POMC)-expressing neurons in the arcuate nucleus, which are themselves glucose sensitive (80).

In a mouse model of nonmetastatic breast cancer, we demonstrated that tumor-bearing mice displayed systemic IL-6 driven inflammation concomitant with hyperglycemia/insulinemia. This was associated with sleep fragmentation, changes in satiety hormone concentrations (leptin/ghrelin), and increased activity of lateral hypothalamic HO neurons, which further drove systemic elevations in glucose via the sympathetic nervous system (10). These findings, in addition to those from mouse models of lung adenocarcinoma (11,81), suggest that IL-6–driven changes in hepatic glucose metabolism indirectly influences central neuromodulator signaling governing arousal and systemic physiology. It remains to be determined whether this mechanism holds true for other neural populations beyond the HO system, however.

## Melanin concentrating hormone

Melanin-concentrating hormone (MCH) neurons also primarily reside in the lateral hypothalamus and are comingled with the HO neurons, although neurons containing MCH mRNA or the neuropeptide itself have been found in the prosencephalon and brainstem (82,83). Broadly, their actions are opposite to those of the hypocretins/orexins. They play a major role in rapid eye movement sleep regulation, and intracerebrovascular (ICV) injections of recombinant MCH dose-dependently increases both nonrapid eye movement and rapid eye movement sleep in rats (84). Additionally, optogenetic stimulation of MCH neurons promotes sleep even during times of heightened arousal (85,86). MCH neurons also serve vital functions in the control of energy expenditure and feeding. Overexpression of MCH promotes hyperphagia and obesity, while mice lacking the MCH peptide or MCH neurons are lean and hypophagic (87-89). In further contrast with adjacent HO neurons, MCH neurons are electrically excited by glucose in the extracellular space (51) and subsequently dictate the nutrient value of ingested sugar (90). Inhibitory interplay between HO and MCH neurons likely allows for fine-tuned control of arousal and feeding behavior through local microcircuitry (85).

How MCH neuronal activity becomes altered in the context of cancer remains largely unexplored. However, as MCH is also expressed in some peripheral tissues, a few studies have examined its role in tumorigenesis and cancer progression. In mouse models of colon adenocarcinoma, mice lacking MCH (total knockout) developed fewer and smaller tumors than those with intact MCH signaling (91). Additionally, Lgr5+ stem cells in the small intestine were observed to express MCH receptors (MCHR1), suggesting that the MCH may drive cancer via its actions on stem cell signaling.

## Proopiomelanocortin

About 50% of POMC neurons located in the hypothalamic arcuate nucleus are directly sensitive to and excited by glucose via the classic β cell pathway, involving ATP-induced closure of K_ATP_ channels containing the Kir6.2 subunit (92). Disruption of glucose sensing in these neurons (via aberrant expression of mutant Kir6.2) causes widespread deficits in systemic glucose homeostasis. Additionally, diet-induced obesity can reduce the glucose-sensing capacity of POMC neurons through a mitochondrial uncoupling protein 2-dependent mechanism (93). These neurons are part of a circuit that suppresses appetite and food intake, along with neurons within the parabrachial nucleus expressing calcitonin-gene related peptide, among others (94,95). In the context of cancer, these cells may play a primary role in cancer cachexia/anorexia, as tumor-induced inflammation can activate these appetite-suppressing neurons to inhibit food intake (96, 97). A more detailed analysis of how these neurons operate in the context of cancer is warranted, as research has highlighted their roles in behavioral problems common among cancer patients, including anxiety (98,99) and depression (100).

## Agouti-related peptide

Arcuate Agouti-related peptide (AgRP) neurons operate in a largely reciprocal fashion to comingled POMC neurons. They sense orexigenic factors such as ghrelin to promote food-seeking behavior (101). They are directly sensitive to extracellular glucose, which has inhibitory actions on neural firing, and AgRP peptide transcription and secretion (102). The effects of glucose on AgRP neural activity seem to be dependent on adenosine 5′-mono-phosphate-activated protein kinase (AMPK), as knocking out this gene in AgRP neurons prevents them from responding to changes in extracellular glucose, but not insulin or leptin (103). AgRP neurons play a major orchestrating role in aligning the timing of food intake with the sleep–wake cycle (104). How cancer-induced changes in glucose concentrations and other orexigenic hormones disrupts AgRP neural activity remains to be determined.

## Neuropeptide Y

Major populations of neuropeptide Y (NPY)-expressing neurons are located in the arcuate nucleus, with another in the nearby lateral hypothalamus that intrinsically sense extracellular glucose and respond to insulin-induced hypoglycemia (105). They operate in a complex network with AgRP, HO, POMC, and MCH neurons to regulate energy balance in response to changes in systemic cues. Similar to AgRP neurons, they are inhibited by glucose in an AMPK-dependent manner (106). In addition to their roles in regulating appetite and food intake, NPY neurons play a significant role in the control of energy expenditure, oxidative fuel selection, and bone metabolism (107). NPY has also been observed to exert anxiolytic actions and function in the manifestation of stress resilience (108). Whether this system plays a role in cancer-associated behavioral comorbidities remains to be determined, although early studies suggest that NPY and its receptors may play a significant role in cancer anorexia (109-111).

## Brainstem–hypothalamus crosstalk

Glucose-sensing neurons in the brainstem coordinate with the hypothalamus to control feeding behavior and energy homeostasis. This was hinted at by studies demonstrating that intrahypothalamic microinjections of norepinephrine inhibits POMC, but activates AgRP, neurons in the arcuate nucleus (112). Additionally, ablation of norepinephrine containing projections to the arcuate (via saporin-conjugated antidopamine beta-hydroxylase injections) alters AgRP and NPY concentrations, leading to impairments in hypoglycemic (glucoprivic) or ghrelin-induced feeding (113,114). Additionally, glucose-sensitive catecholaminergic neurons in the nucleus of the solitary tract promote hypoglycemic feeding via their projections to AgRP neurons in the arcuate (115). Careful examination of parabrachial calcitonin gene-related peptide neurons (which are anorexigenic) in tumor-bearing mice revealed that they are hyperactive in response to cancer in the periphery. Genetic ablation of these neurons, or activation of upstream AgRP neurons, can counteract cancer-induced anorexia (116,117). Examination of brainstem–hypothalamus connections in cancer remains an open area of research that will likely shed light on how changes in peripheral metabolic factors promotes diverse impairments in neuroendocrine and behavioral phenotypes.

## Conclusion

Disrupted metabolism is a hallmark of cancer. Small adjustments in cellular metabolism spurred by oncogene-mediated changes in fuel utilization can lead malignant cells to produce metabolic “waste.” This promotes an inflammatory response from immune cells in the tumor microenvironment which is largely permissive to tumor growth. With sufficient and sustained stimulation, inflammatory signaling molecules (eg, IL-6) can escape into systemic circulation. Actions of cytokines like IL-6 in the liver via classical IL-6Rα/gp130/pSTAT3 signaling promotes the transcription of STAT3 target genes, including its own negative regulator, SOCS3. One consequence of this is ubiquitin-mediated destruction of IRS-1/2, resulting in deficits in glucose uptake and systemic hyperglycemia. Chronic elevations in circulating glucose impair whole-body physiology and aberrantly activate glucose-sensing neurons in the hypothalamus and brainstem. These neural populations have diverse neuroendocrine functions, including the regulation of sleep/wake states, appetite and feeding, and systemic energy balance. Therefore, understanding and combating tumor-induced hyperglycemia may provide significant benefits for both patient quality of life and subsequent mortality.

## Data Availability

Data sharing is not applicable to this article as no datasets were generated or analyzed during the current study.

## Abbreviations

AGE: advanced glycation end product
AgRP: Agouti-related peptide
ATP: adenosine triphosphate
HO: hypocretin/orexin
IRS: insulin receptor substrate
IL: interleukin
LDHA: lactate dehydrogenase A
MCH: melanin-concentrating hormone
NPY: neuropeptide Y
POMC: proopiomelanocortin
RNA: ribonucleic acid
SOCS: Suppressor of cytokine signaling

## Additional Information

***Disclosure Summary:*** The authors report no conflict of interest.
